# An Investigation Into the Impact of Dementia Knowledge and Attitudes on Individuals’ Confidence in Practice: A Survey of Non-Healthcare Staff Inside the Prison Estate in England and Wales

**DOI:** 10.1192/bjo.2022.380

**Published:** 2022-06-20

**Authors:** Sarah Burke, Athanasios Hassoulas, Andrew Forrester

**Affiliations:** Cardiff University, Cardiff, United Kingdom

## Abstract

**Aims:**

Whilst the majority of age groups are seeing a decline in numbers in prison custody, the older male population continues to rise year on year. This unexpected trend has led researchers to investigate the needs of this particular cohort in more detail and start to question if the prison estate is able to care for the specific needs of the ageing population. This primary research specifically, looks to investigate what relationship, if any knowledge and attitudes to dementia have on how the confidence in practice levels of non-healthcare prison staff

**Methods:**

This research, in a specific, applied context considers the relationship between attitudes toward the prison estate alongside knowledge and attitudes toward dementia in general and the potential relationship these may have on confidence levels. To do this, the research scored individuals’ responses against the dementia knowledge assessment scale, attitudes to the prison estate and general attitudes toward dementia. These three independent variables were measured both overall and individually against individual confidence in practices scores. 50 individuals participated with differing roles and length of service in the prison estate

**Results:**

The results of the study found that the overall model was significant. Of the three independent variables, it was found that positive attitudes to dementia were the most influential predictor of confidence. Knowledge of the condition and attitudes to the prison estate, the second and third independent variable however were not significant predictors of confidence in practice levels. Overall, the results indicate that there is a relationship between knowledge, attitudes, and confidence in delivery of dementia care in the prison estate as an overall model.

**Conclusion:**

The main objective of this study was to determine the knowledge and attitudes to dementia of non-healthcare prison staff and if that knowledge and those attitudes had an impact on confidence in practice levels. It could be argued that this research has fulfilled its primary aim, reporting that knowledge about and attitudes toward dementia scores are a significant predictor of knowledge of the condition in non-healthcare-based staff.

